# Complete genomic sequence analysis of infectious bronchitis virus Ark DPI strain and its evolution by recombination

**DOI:** 10.1186/1743-422X-5-157

**Published:** 2008-12-22

**Authors:** Arun Ammayappan, Chitra Upadhyay, Jack Gelb, Vikram N Vakharia

**Affiliations:** 1 Center of Marine Biotechnology, University of Maryland Biotechnology Institute, Baltimore, 701 East Pratt Street, Baltimore, Maryland 21202-3101, USA; 2 Department of Veterinary Medicine, University of Maryland, College Park, MD 20742, USA; 3 Avian Biosciences Center, University of Delaware, 531 South College Avenue, Newark, DE 19716-2150, USA

## Abstract

An infectious bronchitis virus Arkansas DPI (Ark DPI) virulent strain was sequenced, analyzed and compared with many different IBV strains and coronaviruses. The genome of Ark DPI consists of 27,620 nucleotides, excluding poly (A) tail, and comprises ten open reading frames. Comparative sequence analysis of Ark DPI with other IBV strains shows striking similarity to the Conn, Gray, JMK, and Ark 99, which were circulating during that time period. Furthermore, comparison of the Ark genome with other coronaviruses demonstrates a close relationship to turkey coronavirus. Among non-structural genes, the 5'untranslated region (UTR), 3C-like proteinase (3CL^pro^) and the polymerase (RdRp) sequences are 100% identical to the Gray strain. Among structural genes, S1 has 97% identity with Ark 99; S2 has 100% identity with JMK and 96% to Conn; 3b 99%, and 3C to N is 100% identical to Conn strain. Possible recombination sites were found at the intergenic region of spike gene, 3'end of S1 and 3a gene. Independent recombination events may have occurred in the entire genome of Ark DPI, involving four different IBV strains, suggesting that genomic RNA recombination may occur in any part of the genome at number of sites. Hence, we speculate that the Ark DPI strain originated from the Conn strain, but diverged and evolved independently by point mutations and recombination between field strains.

## Findings

Avian infectious bronchitis virus (IBV) is a pathogen of domestic chickens that causes acute, highly contagious respiratory disease [1]. IBV is a member of the *Coronaviridae*, order *Nidovirales *[2] and its genome consists of a 27.6 kb single stranded positive-sense RNA molecule that encodes for four structural proteins; the spike (S) glycoprotein, the small envelope (E) protein, the membrane (M) glycoprotein, and the nucleocapsid (N) protein [3,4]. Six subgenomic mRNAs are transcribed from the IBV genome in virus-infected cells. The mRNA 1 contains two large overlapping open reading frames, encoding two polyproteins 1a and 1b [5], among which 1b is produced as 1ab polyprotein by ribosomal frame-shifting mechanism [6].

Many serotypes have been described for IBV, probably due to the frequent point mutations that occur in RNA viruses and also due to recombination events demonstrated for IBV [7-9]. For this reason, the characterization of virus isolates existing in the field is very important. The Ark DPI strain was first isolated from Delmarva Peninsula broiler flock [10,11] and it is currently being used as a vaccine in the USA and Europe. In this study, we characterized the entire genome of virulent Ark DPI strain (embryo passage 11) and compared it with other IBV strains and coronaviruses from all over the world.

The Ark DPI virus was inoculated into 9-day-old SPF chicken eggs and allantoic fluid was collected 72 h post inoculation. The fluid was clarified by low speed centrifugation and clear supernatant was stored at -80°C. Genomic RNA was extracted from virus-infected allantoic fluid with Qiagen RNAeasy kit, following the manufacturer's instructions, and stored at -80°C until further use. Oligonucleotides were designed based on consensus sequence of the following IBV strains: Cal 99 [GenBank:AY514485], Mass 41 [GenBank:AY851295] and BJ [GenBank:AY319651]. Overlapping primers were designed in a manner such that each pair of primer covered approximately two kb of genome. The RT-PCR was carried out as described earlier [12] and the RT-PCR products were cloned into pCR2.1 TOPO TA vector (Invitrogen, CA). Plasmid DNA from various clones was sequenced by dideoxy chain termination method, using an automated DNA sequencer (Applied Biosystems, CA). Three independent clones were sequenced for each amplicon to exclude errors that can occur from RT and PCR reactions. The assembly of contiguous sequences and multiple sequence alignments were performed with the GeneDoc software [13]. The pair-wise nucleotide identity and comparative sequence analyses were conducted using Vector NTI Advance 10 software (Invitrogen, CA) and BLAST search, NCBI. Phylogenetic analyses were conducted using the MEGA4 program [14].

The GenBank accession number for the Ark DPI sequence is EU418976. The complete genomes of following strains are obtained from GenBank: TCoVMG10, NC_010800; Beaudette, NC_001451; M41, AY851295; CK/CH/LSD/05I, EU637854; A2, EU526388; LX4, AY338732; SAIBK, DQ288927; The accession numbers of IBV gene sequences which are used in this study are as follows: For replicase gene sequences: (a) 5'UTR; Conn, AY392049; Florida, AY392050; CU-T2, AY561724; Ark 99, AY392051; DE072, AY392054;GA98, AY392053; Gray, AY392056; (b) PLpro: Conn, AY392059; Florida, AY392060; CU-T2, AY561734; Ark 99, AY392061; DE072, AY392064; GA98, AY392063; Gray, AY392066 (c) Mpro: Conn, AY392069; Florida, AY392070; CU-T2, AY561744; Ark 99, AY392071; DE072, AY392074; GA98, AY392073; Gray, AY392076; (d) RdRp: Conn, AY392079; Florida, AY392080; CU-T2, AY561754; Ark99, AY392081; DE072, AY392084; GA98, AY392083; Gray, AY392086. For Structural genes (a) Complete structural genes: HK, AY761141; Vic, DQ490221; KB8523, M21515; TW2296/95, DQ646404 (b) S1: Jilin, AY839144; Gray, L18989; Conn, EU526403; Holte, L18988; UK/2/91, Z83976; Qu16, AF349620; JMK, L14070; H120, M21970; GAV-92, AF094817; DE072, AF274435; IS/1366, EU350550; (c) S2: JMK, AF239982; Jilin, AY839146; Holte, AF334685; DE072, AY024337; Conn, AF094818; Gray, AF394180; H120, AF239982; (d) S: Ark 99, L10384; CU-T2, U04739; (e) Gene 3: Jilin, AY846833; Conn, AY942752; CU-T2, U46036; Ark 99, AY942751; Gray, AF318282 (f) M: Jilin, AY846833; JMK, AF363608; Conn, AY942741; H120, AY028295; Gray, AF363607; (g) Gene 5: Jilin, AY839142; Gray, AF469011; Conn, AF469013; DE072, AF203000; (h) N: Jilin, AY839145; Ind/TN/92/03, EF185916; Conn, AY942746; H120, AY028296; Gray, M85245; (i) 3'UTR: H120, AJ278336.

The genome of Ark DPI consists of 27,620 nucleotides (nts), excluding poly (A) tail, and comprises ten open reading frames (ORFs) flanked by 5' (528 nts) and 3' (507 nts) untranslated regions (UTRs). The genome organization is ORF1ab (529–20360), ORF2 (20311–23820), ORF3abc [3a, (23820–23993), 3b (23993–24187), 3c (24168–24491)], ORF4 (24469–25140), ORF5ab [5a (25500–25697), 5b (25694–25942)] and ORF6 (25885–27114).

**Figure 1:**
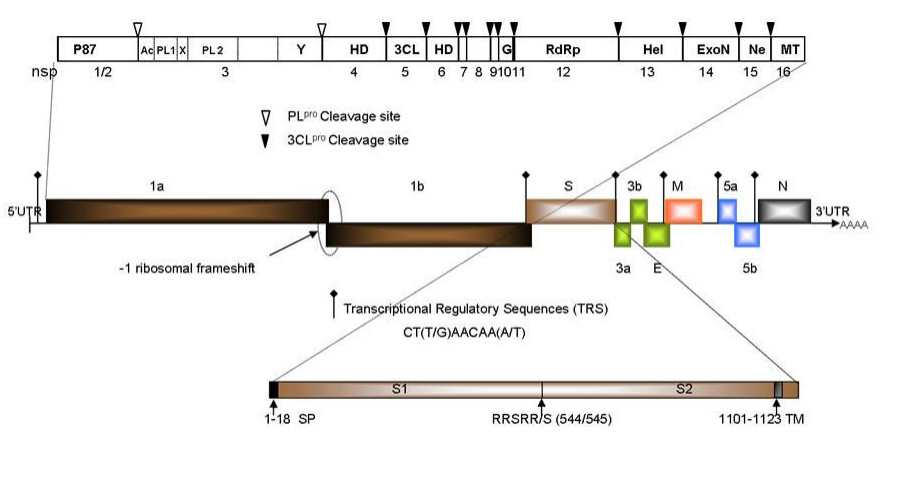
**Classical Genome Organization of IBV-Ark DPI**. The genome of Ark DPI is 27,620 nt long, excluding poly (A) tract. Middle: ten genes and its ORFs. Ribosomal frameshift and position of transcriptional regulatory sequences (TRS) of each gene is indicated. Top: putative domains of ORF1a/1b polyprotein: nsp-non-structural protein, Ac-acidic domain, X-unknown domain X, PL1- papain like proteinase1, PL2-papain like proteinase 2; Y-unknown domain Y; HD-hydrophobic domain, 3CL-3C-like proteinase, G-Growth factor like protein, RdRp-RNA dependent RNA polymerase, Hel-helicase, ExoN-exoribonuclease, Ne-nidoviral uridylate-specific endoribonuclease, MT- 2'-O-ribose methyltransferase. Bottom: details of spike protein; SP-signal peptide, RRSRR/S- spike protein cleavage site between 544 and 545aa, TM-transmembrane domain of spike protein.

**Figure 2:**
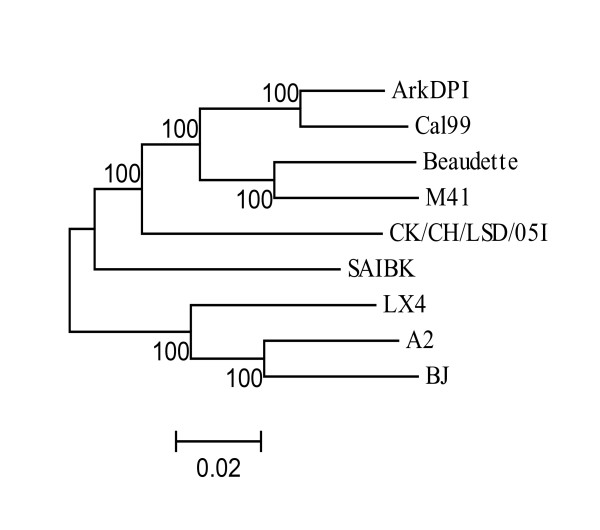
**Phylogenetic tree analysis of complete Ark DPI genome sequence with other IBV strains**. Phylogenetic tree analysis was conducted by neighbor-joining method using bootstrap analysis (100 replications). The scale at the bottom indicates the number of substitution events.

The details of genome organization of Ark DPI are shown in Fig. 1. IBV polyprotein is cleaved into 15 cleavage products, among which first two N-terminal products are cleaved by PL^pro ^and rest of the C-terminal products are cleaved by 3CL^pro ^[15]. The putative domains and their cleavage sites (Fig. 1) are predicted by comparison of amino acid sequences of each non-structural protein (nsp) of Ark DPI with those of IBV-Beaudette which is available in Coronavirus Database (CoVDB) [16]. The nucleotide and the amino acid identity of Ark DPI with other IBVs and coronaviruses are listed in Tables 1, 2, 3. The whole structural gene of Jilin is 100% identical to Ark DPI, which suggests that Jilin strain is actually Ark DPI, which is currently used as a vaccine in China [17]. The whole genome comparison of IBV strains reveals a close relationship of Ark DPI with Cal 99 (96% identity), as shown in Fig. 2. Earlier studies have shown that Cal 99 probably evolved from Ark DPI [18].

**Table 1:** Percent (%) nucleotide identity of Ark DPI non-structural genes and ORF1ab, ORF2-6 and complete genome with other IBV strains^a, b, c^

IBV Strains	5'UTR	PL^pro^	M^pro^	RdRp	ORF1	ORF2-6	Complete genome
**Ark99**	**98**	87	**99**	**99**	NA	**96**	NA

A2	95	83	85	**99**	86	85	86

Beaudette	**96**	85	93	93	91	91	91

BJ	**96**	83	88	93	86	85	86

Cal99*	**100**	**99**	**97**	**99**	**96**	94	**96**

CK/CH/LSD/05I	**97**	84	88	90	89	90	89

**Connecticut**	**97**	87	**100**	**99**	NA	**96**	NA

CU-T2*	**100**	94	**99**	**99**	NA	94	NA

DE072	**100**	**100**	90	90	NA	NA	NA

Florida	**97**	87	**100**	**99**	NA	NA	NA

GA98*	**100**	**100**	**100**	**100**	NA	NA	NA

**Gray**	**100**	87	**100**	**100**	NA	NA	NA

Jilin	NA	NA	NA	NA	NA	**100**	NA

LX4	94	83	85	89	87	84	86

M41	**97**	87	91	94	91	91	91

SAIBK	92	85	90	90	90	86	89

^a ^Sequences with > 95% identity are in bold letters

^b ^NA-not analyzed

^c ^Parental strains of Ark DPI are shown in bold letters and immediate derivative of Ark DPI is indicated by asterisk (*).

**Table 2:** Percent (%) nucleotide identity of Ark DPI structural genes with other IBV strains ^a, b, c, d^

BV Strains	S1	S2	3a	3b	3c	M	5a	5b	N	3'UTR
**Ark99**	**97(96)**	**96(95)**	92	**99**	**96(92)**	**97(97)**	93	**97**	**98(98)**	**97**

Beaudette	81(79)	95(94)	91	84	88(83)	91(93)	85	93	93(95)	**97**

BJ	77(75)	85(89)	88	76	87(79)	90(93)	NA	NA	89(93)	87

Cal99*	87(84)	94(93)	92	**99**	94(90)	**97(96)**	**100**	**98**	**95(98)**	89

CK/CH/LSD/05I	78(75)	88(91)	89	84	(87)	**96(96)**	**99**	**100**	**100(99)**	90

**Conn**	81(77)	**96(96)**	92	**99**	**100(100)**	**100(100)**	**100**	**100**	**100(100)**	NA

CU-T2*	**96(94)**	93(93)	88	98	92(87)	88(87)	**97**	**99**	**95(96)**	**97**

DE072	62(50)	75(76)	NA	NA	NA	NA	**98**	**98**	NA	**98**

**Gray**	83(80)	**99(99)**	NA	NA	**96(92)**	**98(98)**	**97**	**99**	**97(97)**	**98**

GAV-92	94(92)	NA	NA	NA	NA	NA	NA	NA	NA	NA

H120	81(78)	NA	NA	NA	NA	92(94)	NA	NA	**93(96)**	83

HK*	81(78)	**96(96)**	**99**	**100**	**100(100)**	**100(100)**	**100**	**100**	**100(100)**	NA

Holte	83(80)	95(95)	NA	NA	NA	NA	NA	NA	NA	NA

Ind/TN/92/03	NA	NA	NA	NA	NA	NA	NA	NA	92(94)	NA

IS/1366	78(75)	NA	NA	NA	NA	NA	NA	NA	92(95)	NA

Jilin*	**100(99)**	**100(100)**	**100**	**100**	**100(100)**	**100(100)**	**100**	**100**	**100(100)**	**100**

**JMK**	84(82)	**100(100)**	NA	NA	NA	**97(98)**	NA	NA	NA	NA

KB8523	81(78)	91(92)	NA	NA	NA	93(95)	NA	NA	**95(96)**	NA

LX4	77(76)	85(88)	86	76	88(80)	91(91)	82	90	89(93)	NA

M41	81(78)	95(94)	91	85	88(83)	91(95)	90	**97**	94(95)	**97**

Qu16	84(81)	NA	NA	NA	NA	NA	NA	NA	NA	NA

SAIBK	79(77)	87(91)	86	83	85(80)	89(93)	84	**96**	87(92)	88

TW2296/95	79(77)	86(90)	86	85	(83)	91(92)	82	**96**	89(92)	NA

UK/2/91	78(76)	NA	NA	NA	NA	NA	NA	NA	NA	NA

Vic	81(79)	89(92)	88	88	88(88)	89(95)	87	94	90(94)	NA

^a ^Sequences with > 95% identity are indicated in bold letters

^b ^Amino acid sequences within the parenthesis

^c ^NA-Not Analyzed

^d ^Parental strains of Ark DPI are shown in bold letters and immediate derivative of Ark DPI is indicated by asterisk (*).

**Table 3:** Percent (%) amino acid identity of Ark DPI replicase and structural proteins to other coronaviruses ^c, d^

Coronaviruses^a^	3CL^pro^	**RdRp**	S	E	M	N	Complete genome^b^
BatCoV	40	**70**	22	11	29	25	46

BCoV	41	**67**	21	13	30	24	47

ECoV	41	**67**	22	13	30	25	47

FCoV	45	**62**	22	16	23	23	48

HCoV 229E	40	**63**	23	12	26	23	49

TGEV	46	**61**	22	20	25	26	49

MHV A59	40	**69**	21	14	31	26	46

SARS CoV	46	**68**	21	17	29	22	45

SW1	**56**	**79**	25	28	36	35	50

**TCoV**	**96**	**98**	34	**91**	**97**	**95**	**88**

^a ^BatCoV, Bat coronavirus; FCoV, feline coronavirus; HCoV, human coronavirus; BCoV, bovine coronavirus; MHV, mouse hepatitis virus; SARS-CoV, severe acute respiratory syndrome coronavirus; SW1, beluga whale coronavirus; TCoV, turkey coronavirus.

^b ^Percent nucleotide identity of entire genome

^c ^Sequences with > 50% identity are in bold letters

^d ^Gene in bold letters (RdRp) is highly conserved; TCoV exhibits significant identity with IBV-Ark DPI (marked in bold letters).

The complete genome sequence analysis of Ark DPI strain shows striking similarity to the Conn, Gray, JMK, and Ark 99 IBV strains, which were circulating during that time period [1,19-21]. The 5'UTR, PL^pro^, M^pro ^and RdRp sequence analysis demonstrates that Ark DPI is 100% identical to Gray strain, except for PL^pro ^which has 87% identity, as shown in Table 1. It was suggested that PL^pro ^gene has high genetic variation because of selection pressure [22]. From this analysis, it appears that genetic mutation may have occurred at PL^pro ^gene level. The modern strain GA98 maintains 100% identity with Ark DPI in replicase proteins and because of unavailability of sequence information for rest of the genome; we speculate that GA98 may be a derivative of the Ark DPI strain.

**Figure 3:**
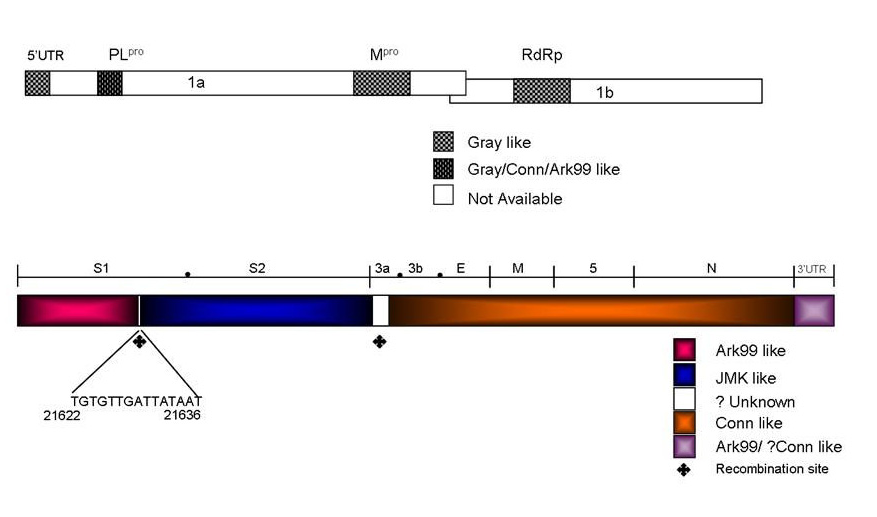
**Schematic presentation of the structural region of Ark DPI genome**. Entire genome of Ark DPI was analyzed for its similarity with other IBV strains. Top panel: 5'UTR & ORF1. Shadowed regions were used for comparative analysis. 5'UTR-5'-untranslated region; PL1-papain like proteinase1; M^pro^-main or 3C-like proteinase; RdRp-RNA-dependent-RNA polymerase. Bottom panel: ORF2 to 3'UTR. Structural genes and their ORFs are marked by (●). Conserved sequence TGTGTTGATTATAAT in S1 gene is shown; ◆ denotes plausible recombination site in Ark DPI.

Analysis of the structural region of Ark DPI clearly demonstrates that it is a chimera of three strains. The S1 gene of Ark DPI is probably derived from Ark 99 (97% identical) and because of genetic mutations in the S1 region, Ark DPI may have evolved independently. There is an A-T rich sequence TGTGTTGATTATAAT (Fig. 3) at the 3'terminus of S1 gene (~300 nts upstream from the end of S1 gene) which is conserved among most of the IBV strains. The S1 gene of Ark 99 maintains its identity with Ark DPI up to this conserved region, but from this point onwards to the end of S2, the nucleotide sequence is 100% identical to JMK strain. The recombination between JMK and Ark 99 had taken place presumably between above mentioned conserved region and intergenic (IG) region of S gene, which is located 49 nts upstream of start codon of S gene. It is speculated that IG sequences serve as "hot spots" for recombination because of its consensus nature [23]. Gray and JMK strains share 99% homology both in the S1 and S2 genes of Ark DPI, but JMK shows greater identity than Gray strain, as shown in Table 2. It is interesting that very few residues in the S1 gene make the Gray strain nephrotropic, whereas JMK is pneumotropic [24].

Out of 174 nts of gene 3a of Ark DPI, last 74 nts are 100% identical to Conn, whereas first 100 nts are only 86% identical. Even though it is not clear whether the 5'-end of 3a was derived from Conn or JMK, but it is evident that the recombination event may have occurred between JMK and Conn at gene 3a. The 3b gene of Ark DPI and Conn differed only by two nucleotides and both share 99% identity, suggesting that 3b belongs to Conn strain. From gene 3c to N gene, Ark DPI shares 100% identity with Conn. It is obvious that the entire structural genome, except spike, belongs to Conn strain. Cross protection studies carried out by Gelb and coworkers [11] demonstrated that the birds immunized with Ark DPI showed 95%, 90% and 63% protection against Conn, Ark 99 and JMK strains, respectively. Indeed, these results suggest that major part of Ark DPI genome was derived from Conn. The level of protection for JMK is 80%, when Ark 99 was used as immunogen [25]. On the other hand, Conn and JMK immunization induces inadequate immunity against Ark-type IBV challenge, suggesting that Ark cross-immunity to JMK and Conn is a one-way relationship [10,11,26].

Recombination hot spots have been demonstrated for IBV isolates by many researchers. These hot spots have been detected in the IG region [23], S1 gene [27], 3' terminus of S2, N and between N gene and 3'UTR [8,28]. Some earlier sequencing studies had provided circumstantial evidence of recombination events in field isolates of IBV [7,29,30]. More or less recombination sites were detected over the entire genome of coronavirus [31]. Based on these results, we speculate that the Ark DPI strain originated from the Conn strain, but diverged and evolved independently by point mutations and recombination between field strains. These findings suggest that there is high level of genetic diversity among currently circulating IBV serotypes. Most of them come from genetic changes which already exist in the IBV field strains and from IBV live vaccines. So frequent monitoring is highly essential to track the emergence of new variants and is mandatory to develop efficient vaccination strategies to control and prevent IB.

## Competing interests

The authors declare that they have no competing interests.

## Authors' contributions

VNV and JG conceived the study. AA planned the experimental design, AA and CU carried out cloning and sequencing. AA drafted the manuscript. All authors critically reviewed and approved the final manuscript.
